# Changes in chemotherapy-induced cognitive impairment in gastrointestinal cancer survivors using multidomain assessments: a prospective cohort study

**DOI:** 10.1007/s11764-025-01759-8

**Published:** 2025-02-15

**Authors:** Kazuya Saita, Kazuaki Tanabe, Yoichi Hamai, Masami Yamauchi, Fumiko Kaneko, Yukio Mikami, Wataru Okamoto, Morihito Okada, Hideki Ohdan, Hitoshi Okamura

**Affiliations:** 1 Department of Psychosocial Rehabilitation, Graduate School of Biomedical and Health Sciences, Hiroshima University, 1-2-3, Kasumi, Minami-Ku, Hiroshima, 734-8551 Japan; 2 Department of Perioperative and Critical Care Management, Graduate School of Biomedical and Health Sciences, Hiroshima University, Hiroshima, Japan; 3 Department of Gastroenterological and Transplant Surgery, Hiroshima University, Hiroshima, Japan; 4 Department of Surgical Oncology, Hiroshima University, Hiroshima, Japan; 5 Department of Clinical Oncology, Hiroshima University Hospital, Hiroshima, Japan; 6 Department of Clinical Oncology, Hiroshima Prefectural Hospital, Hiroshima, Japan; 7 Department of Rehabilitation Medicine, Hiroshima University Hospital, Hiroshima, Japan

**Keywords:** Cancer-related cognitive impairment, Prefrontal cortex, Neuroimaging, Cognitive function, Near-infrared spectroscopy

## Abstract

**Purpose:**

Risk factors for cancer-related cognitive impairment (CRCI) are diverse; neuroimaging instruments are recommended to complement subjective and objective cognitive assessments. This study aimed to evaluate the feasibility of a multidomain assessment protocol for CRCI in gastrointestinal cancer survivors.

**Methods:**

Twenty-four patients with gastrointestinal cancer were scheduled for chemotherapy, and 24 healthy controls were recruited. The Functional Assessment of Cancer Therapy-cognitive function (FACT-Cog) was used to assess subjective cognitive functions. Objective cognitive function was assessed using the trail making test, auditory verbal learning test (AVLT), and verbal fluency test. Cerebral hemodynamic changes in the prefrontal cortex were measured using portable functional near-infrared spectroscopy (P-NIRS). Assessments were conducted at baseline and 6-month follow-up.

**Results:**

Thirty-eight participants were included in the analysis. There was a statistically significant difference in AVLT-delayed recall (*p* = 0.002) in the chemotherapy group compared with the healthy control group, but no significant difference in either group for other cognitive assessments. The chemotherapy group exhibited reduced activity in the left frontal pole at 6 months post-treatment compared to baseline (*p* = 0.018).

**Conclusions:**

Gastrointestinal cancer survivors who receive chemotherapy may exhibit poorer delayed recall of memory functions than healthy individuals. Monitoring prefrontal cortical hemodynamics using P-NIRS during cognitive tasks is feasible for clinical application and understanding CRCI symptoms.

**Implications for Cancer Survivors:**

These multidomain assessments are translatable to clinical practice and useful for other cancers. Additionally, the P-NIRS assessments may offer a deeper understanding on the impact of depressive symptoms and declining motivation on the cognitive function of cancer survivors.

**Supplementary Information:**

The online version contains supplementary material available at 10.1007/s11764-025-01759-8.

## Introduction

Global cancer incidence is estimated to increase by 20 million new cases in 2022, half of which will occur in Asia [1]. In Japan, the number of cancer survivors is estimated to reach approximately 3.6 million, with a prevalence rate of 36% by 2050 [2]. One problem with cancer survivorship care is cancer-related cognitive impairment (CRCI) [3]. Chemotherapy-induced cognitive dysfunction is a major impairment experienced by cancer survivors during the treatment process and is also known as *Chemobrain* [4, 5]. The degree of impairment in CRCI is similar to that in mild cognitive disorders. Specific cognitive domains include attention, processing speed, memory, and executive function [6, 7]. Several factors are thought to be associated with the occurrence of CRCI, including the cancer itself, effects of cancer treatment (e.g., chemotherapy, hormone therapy), co-occurring symptoms (e.g., fatigue, distress), genetic factors (e.g., APOE-4), physiological factors (e.g., dysregulation of inflammatory cytokines, disruption of the blood–brain barrier), and sociodemographic factors (age, education level) [8, 9]. These factors complicate our understanding of CRCI symptoms. While CRCI has been extensively studied in breast cancer survivors [5, 7], evidence regarding its effects on gastrointestinal cancer survivors remains limited. Gastrointestinal cancers, including colorectal, gastric, and esophageal cancers, rank among the most prevalent cancers globally, irrespective of sex. These cancers are particularly significant in East Asia, including Japan, where lifestyle and environmental factors contribute to their high incidence rates [10]. This study specifically examines gastrointestinal cancer survivors to address the paucity of data on neurocognitive changes in this group. Investigating the effects of chemotherapy on neurocognition in these survivors is critical for improving survivorship care and enhancing their quality of life.

The recommendations of the International Cognition and Cancer Task Force (ICCTF) include several statements for study designs to capture CRCI: (1) longitudinal studies that include pre- and post-treatment, (2) disease-specific and healthy control groups, and (3) neuroimaging data for objective and subjective assessment [11]. Regarding assessment methods for CRCI, the ICCTF has recommended the following neuropsychological tests (NPTs) [6]: the trail making test (TMT) [12], Hopkins Verbal Learning Test-Revised [13], and Controlled Oral Word Association Test (COWA) [14]. Nevertheless, 10 years after their advocacy, these recommended assessments are not being used worldwide for CRCI assessment, and a consensus is yet to be established [7]. This may be because although NPTs are relatively widespread worldwide, the assessments are language-mediated and have not been standardized in minority language-speaking countries. Similar verbal memory and word fluency tests are available in all countries; hence, reports from minority-language countries are necessary to understand the incidence of CRCI. On the other hand, patient-reported outcomes (PROs) as subjective assessments are also sensitive to CRCI detection. The Functional Assessment of Cancer Therapy-Cognitive (FACT-Cog) version 3 is the most frequently used international assessment of PRO assessing CRCI [15].

In addition to these CRCI assessments, neurophysiological biomarkers measuring brain activity are recommended as adjunct diagnostic tools [16]. Several neuroimaging devices are used to measure brain activity, and functional magnetic resonance imaging (fMRI) is a representative device [7]. However, given its clinical application, applying fMRI to all non-central nervous system cancers is not feasible from an economic and time-effective perspective. Functional near-infrared spectroscopy (fNIRS) is a noninvasive neuroimaging method that addresses this problem [17]. fNIRS measures changes in cerebral blood flow associated with neural activity based on the neurovascular coupling (NVC) response [18]. The fNIRS device is expected to bridge the gap between NPT and PRO as an adjunct diagnostic tool for current cognitive assessment [16, 17]. In this study, we applied portable fNIRS (P-NIRS), which is less restraining and burdensome to patients and can be used under conditions similar to those in daily life. To our knowledge, only one study has reported the clinical application of fNIRS measurements in addition to NPT and PRO to detect CRCI in breast cancer [19], and none has reported on the use of the P-NIRS device in gastrointestinal cancer.

This study aimed to prospectively investigate neurocognitive changes in gastrointestinal cancer survivors before and after chemotherapy using a multidomain assessment and compare them with healthy volunteers. The assessment encompassed both subjective and objective measures of cognitive function across various domains, such as memory and executive function, supplemented with neuroimaging data. To capture neurocognitive changes from multiple perspectives, the secondary aim was to investigate the feasibility of clinical applications for measuring cortical hemodynamic changes based on NVC responses as an additional diagnostic tool in conjunction with cognitive assessments.

## Materials and methods

### Study design and participants

This study prospectively recruited 48 volunteers to enroll between September 2022 and December 2023, 24 each in the cancer chemotherapy (C +) and healthy control (HC) groups. Each participant was enrolled after eligibility checks with written consent after providing informed consent for the study. Measurements were completed before chemotherapy or baseline assessments and then after 6-month follow-up assessments and were evaluated under the same environmental conditions. Based on previous neuroimaging studies [20, 21], we estimated the feasible number of participants from previous outpatients at our single institution and determined a sample size of 20 for the C + group. To account for a potential 20% dropout rate, we increased the sample size estimate to 24 for the C + group. Similarly, with 24 cases in the age- and sex-matched HC group, a total of 48 cases were included in the study. This study was approved by the Ethical Review Committee for Epidemiological Research of Hiroshima University (E2022-0061).

The C + group included outpatients at the Hiroshima University Hospital who were to begin their first course of chemotherapy following a diagnosis of gastrointestinal cancer (colorectal, gastric, or esophageal cancer). The inclusion criteria for the C + group were as follows: (1) individuals who were at least 20 years and < 85 years old, (2) individuals who met the diagnostic criteria for gastrointestinal cancer, (3) individuals who were scheduled to start the first course of chemotherapy treatment, (4) individuals with no anemic symptoms and a hemoglobin level of > 8.0 g/dL on a blood test, (5) individuals with an Eastern Cooperative Oncology Group-performance status score (ECOG-PS) of 0 or 1, (6) individuals whose first language is Japanese, (7) individuals scheduled for outpatient visits for at least 6 months, and (8) individuals who provided written consent to participate in this study. The exclusion criteria were as follows: (1) individuals with a history of chemotherapy treatment for cancer, (2) individuals with a diagnosis of dementia or cognitive decline equivalent to dementia (Mini-Mental State Examination, Japanese version (MMSE-J) [22, 23], less than 24 points), (3) individuals with a diagnosis of severe depression or psychiatric disorder, (4) individuals regularly using sleeping pills due to sleep disorders, (5) individuals with primary brain tumors, metastases, or disorders to the central nervous system, (6) individuals with a history of alcohol or drug addiction, (7) individuals with a history of opioid analgesic use due to cancer pain, (8) individuals with cachexia or other poor systemic conditions, (9) individuals with serious liver disease, renal disease, seizure disorder, cardiac complications, (10) pregnant and lactating women, and (11) other individuals judged to be inappropriate by the principal investigator. The HC group recruited healthy volunteers from staff or family members of patients in outpatient rehabilitation facilities and community centers in the same residential area. The inclusion criteria for the HC group were as follows: (1) individuals who were at least 20 years and < 85 years old, (2) independent in activities of daily living (Barthel Index > 85 points), (3) individuals whose first language is Japanese, (4) individuals live in a permanent residence and can be examined after 6 months, and (5) individuals who provided written consent to participate in this study. The exclusion criteria for the HC group were as follows: (1) individuals with a history of cancer diagnosis and chemotherapy treatment for cancer, (2) individuals with a diagnosis of dementia or cognitive decline equivalent to dementia (MMSE-J, less than 24 points), (3) individuals with a diagnosis of severe depression or psychiatric disorder, (4) individuals regularly using sleeping pills due to sleep disorders, (5) individuals with a history of cerebrovascular disease (stroke, neurodegenerative disease), (6) individuals with a history of alcohol or drug addiction, (7) individuals with a history of opioid analgesic use due to pain, (8) individuals with serious liver disease, renal disease, seizure disorder, cardiac complications, (9) pregnant and lactating women, and (10) other individuals judged to be inappropriate by the principal investigator. The systemic condition and medical history of the C + group were collected using information from electronic medical records or self-reported by the individuals; information for the HC group was collected by self-report from the individuals. It was planned to exclude from the analysis cases where follow-up data could not be obtained due to an individual’s unanticipated life events or individual intent. Individuals for whom follow-up data were not available due to unforeseen life events were planned to be excluded from the analysis.

### Assessments and fNIRS measurements

During the eligibility check, the following information was collected from the individuals as sociodemographic factors related to cognitive function: age, sex, education level, marital status, and employment status. Clinical characteristics, including tumor stage, cancer site, line of therapy, and chemotherapy regimens, were collected from the electronic medical records at our facility. In both groups, the MMSE-J and the Japanese version of the National Adult Reading Test [24, 25] were used to assess cognitive functional screening and predict IQ as a cognitive reserve at baseline. In the C + group, the co-occurring symptoms of cancer-related fatigue, sleep disturbances, and pain were investigated through self-reporting; anemia was screened based on blood hemoglobin levels.

The Japanese version of FACT-Cog version 3 [15, 26] was used to assess subjective cognitive complaints. The FACT-Cog is a 37-item, 5-point questionnaire that assesses perceived cognitive decline and recommends the use of sub-items rather than the total score, as it is divided into four sub-items. The study used scores for perceived cognitive impairments (CogPCI: score range, 0–72) and perceived cognitive abilities (CogPCA: score range, 0–28).

The Japanese versions of the TMT (TMT-J) [12, 27], Rey auditory verbal learning test (AVLT) [28, 29], and verbal fluency test (VFT) [14, 30] were used to evaluate objective cognitive function. The performance time of the TMT-J was assessed for part A (a task to connect numbers in sequence) to measure the processing speed domain and part B (a task to connect numbers and letters alternately) to measure the executive function domain. The AVLT was used to assess the verbal memory domain, and the following main outcome measures were assessed: total number of words from the first to fifth (AVLT-sum) and number of delayed recalls (AVLT-DR/AVLT-7). A letter fluency task was selected for the VFT to assess the language and executive function domains. The total number of 3 mora generated per 60 s was recorded according to COWA, and the number of generated words and duplicate errors for each mora (/a/ /ka/ /shi/) were recorded.

Continuous-wave P-NIRS (HOT-2000, NeU Corporation, Tokyo, Japan) is a wireless device with a Bluetooth connection. The sampling rate was 10 Hz, and the wavelength of the near-infrared light was 810 nm. This device allows the measurement of changes in total hemoglobin (THb) concentration according to the modified Beer–Lambert law; the reliability of prefrontal cortex measurements compared to multichannel fNIRS has been demonstrated by prior P-NIRS devices [31]. The NVC response is the principle of fNIRS measurements, and THb is one of the representative indicators of fNIRS, which represents the sum of oxyhemoglobin (O_2_Hb) and deoxyhemoglobin (HHb) concentrations. In a typical NVC reaction, THb and O_2_Hb concentrations increase if HHb remains unchanged. The P-NIRS instrument incorporates a multi-distance optodesis method [32] that removes the skin blood flow component by implementing a short channel to compensate for the shortcomings of previous fNIRS studies [33]. Based on the International 10–20 method, the positions of the left and right channels were defined as the positions corresponding to the frontal pole (FP) on the Fp1 and Fp2 lines or the lateral region (corresponding to part of the ventral and dorsal prefrontal cortex) as the lateral prefrontal cortex (LPFC). The participants in the P-NIRS experiment were seated in a quiet environment. The experiment used a block design method, and the most commonly used phonemic fluency task was the cognitive task for the fNIRS experiment. Changes in cortical activity during a phonemic fluency task are known to be highly relevant to the prefrontal cortex measured as regions of interest [34]. Based on previous studies [35], a modified protocol was used, in which, after an initial 30-s rest period, a 120-s cycle consisting of a 30-s control task, a 60-s phonemic fluency task, and a 30-s recovery task was repeated three times. The control task consisted of repeated vocalizations of the Japanese vowels (/a/, /i/, /u/, /e/, and /o/). The VFT comprised two sets of three 60-s repetitions of one more as a phonemic fluency task (/shi/, /i/, /re/, or /a/, /fu/, /ni/).

### Analysis

Representative values for sociodemographic data, clinical characteristics, and cognitive functional assessment are expressed as means or medians, depending on the skewness of the distribution of the descriptive data. The Shapiro–Wilk test was used to test for normality. To compare the baseline characteristics and clinical data between the groups, we used the two-sample *t*-test or Wilcoxon rank-sum test for continuous variables and the chi-square test for categorical variables. Several parameters of the objective cognitive assessment using NPTs were transformed into age-matched normalized scores (*Z*-score) based on previous studies to obtain consistent descriptive data [27, 29, 30]. The TMT-A, TMT-B, and VFT errors were flipped values of the *Z*-score used so that the positive direction of cognitive performance had a better score. Welch’s two-sample *t*-test was used to compare differences in changes between the groups in each cognitive function assessment. Missing data without follow-up assessments were excluded. To compare within-group data, the paired *t*-test or Wilcoxon signed-rank test was used. To calculate the percentage of CRCI incidence, PRO and NPT were based on the following methods: for NPT, the ICCTF-recommended criteria for CRCI were used, defined as ≥ ±1.5 SD of the standard for two NPTs or ≥ ±2.0 SD for one NPT [6]; for PRO by FACT-Cog, the clinically important difference in FACT-Cog was defined as subscale scores ranging from 6.4 to 10.3%, so we defined this as a decrease of at least 4.6 points (6.4% decrease) on the CogPCI score or at least 1.8 points (6.4% decrease) on the CogPCA [36].

For the fNIRS analysis, pre-processing of the raw data was performed using the statistical analysis software TOMATO (HOT-ANALYSIS; NeU Corporation, Tokyo, Japan). A median filter and moving average were used for noise reduction. First, the median filter was set to a time window of 1 s to reduce the spike noise caused by body movement. Subsequently, the moving average was set to 3 s to smoothen the waveform data. The smoothed waveform data were averaged, and a baseline correction was performed with the task start time set to zero. To allow for inter-channel and inter-subject comparisons, we converted the *Z*-score to represent the change in THb concentration. It was converted to a normalized averaging waveform by dividing by the standard deviation of the 20 s value of the control task. Matlab R 2023a (Mathworks, Inc., Natick, MA, USA) was used to analyze the fNIRS data after pre-processing. Linear mixed models to account for repeated measures were used for between-group and within-group comparisons of the fNIRS data. The model included random effects for each individual participant, while fixed effects included group (C+ and HC), period (baseline and follow-up), and task condition (control task and VFT task). Variance components were estimated using restricted maximum likelihood methods. The two-sided significance level was set at 5%. All statistical analyses were performed using JMP pro 17.0.0 (SAS Institute Inc., Cary, NC, USA).

## Results

After eligibility checks, 48 participants (24 volunteers each in the C + and HC groups) were enrolled, and 38 participants (19 in each group; median age, 74 years; 12 men and 7 women, respectively) were finally included in this study. Of the five individuals in the C + group who dropped out, four died, and one was difficult to follow up due to outpatient treatment outside the prefecture. Of the five individuals in the HC group who dropped out, two had surgery due to orthopedical conditions, and two were hospitalized due to cardiac and cerebrovascular diseases and, therefore, could not undergo follow-up assessments. The other did not wish to be followed up due to the bereavement of a family member. Baseline sociodemographics and clinical characteristics are presented in Tables 1 and 2.

**Table 1:** Between-group comparisons of baseline sociodemographic characteristics and cognitive assessments

	Total (*N* = 38) Mean [95% CI]	C + group (*N* = 19) Mean [95% CI]	HC group (*N* = 19) Mean [95% CI]	*p*-value
Sociodemographic characteristics				
Age				
Median (IQR)	74 (64–79.25)	74 (67–77)	74 (49–80)	0.977^b^
Sex				
Male (%)	24 (63.2)	12 (63.2)	12 (63.2)	1.000^c^
Female (%)	14 (36.8)	7 (36.8)	7 (36.8)	
Race/ethnicity				
Asian/Japanese (%)	38 (100.0)	19 (100.0)	19 (100.0)	1.000^c^
Education level				
High school (12 ≧) (%)	23 (60.5)	13 (68.4)	10 (52.6)	0.319^c^
College (12 <) (%)	15 (39.5)	6 (31.6)	9 (47.4)	
Marital status				
Single (%)	5 (13.2)	3 (15.8)	2 (10.5)	0.631^c^
Married (%)	33 (86.8)	16 (84.2)	17 (89.5)	
Employment status				
Unemployed (%)	22 (57.9)	14 (73.7)	8 (42.1)	0.049^c*^
Being employed (%)	16 (42.1)	5 (26.3)	11 (57.9)	
Objective cognitive assessments				
MMSE, score, median (IQR)	30 (28–30)	30 (29–30)	30 (28–30)	0.772^b^
JART, FSIQ	102.58 [99.11, 105.89]	100.21 [94.98, 105.45]	104.79 [100.23, 109.35]	0.175^a^
TMT-A, sec, median (IQR)	47 (33–63.25)	43 (33–74)	51 (34–59)	0.759^b^
TMT-B, sec, median (IQR)	76.5 (60.5–103)	78 (59–119)	75 (67–98)	0.781^b^
AVLT-sum, words	38.92 [35.55–42.29]	37.11 [32.07, 42.14]	40.74 [35.93, 45.55]	0.281^a^
AVLT-DR, words	7.58 [6.51, 8.65]	7.32 [5.65, 8.99]	7.84 [6.36, 9.33]	0.624^a^
VFT-sum, words	32.84 [29.09, 36.60]	32.89 [27.83, 37.96)	32.79 [26.74, 38.84]	0.978^a^
VFT-error, words	1.97 [1.27, 2.68]	2.42 [1.59, 2.42]	1.52 [0.34, 2.71]	0.202^a^
Subjective cognitive assessments, FACT-Cog				
Cog PCI, score, median (IQR)	65.5 (60.5–70)	66 (59–70)	65 (61–68)	0.568^b^
Cog PCA, score, median (IQR)	23 (22–25)	24 (22–26)	23 (22–25)	0.385^b^

*AVLT-DR*, (Rey) Auditory Verbal Learning Test—delayed recall (Trial 7); *AVLT-sum*, (Rey) Auditory Verbal Learning Test—sum (Trial 1–5); *C* + *group*, cancer chemotherapy group; *CI*, confidence interval; *Cog-PCA*, cognitive function – perceived cognitive ability; *Cog-PCI*, cognitive function – perceived cognitive impairment; *FACT-Cog*, Functional Assessment of Cancer Therapy—Cognitive Function; *FSIQ*, full-scale intelligence quotient; *HC group*, healthy control group; *IQR*, interquartile range; *JART*, Japanese Adult Reading Test; *MMSE*, Mini-Mental State Examination; *TMT-A*, Trail Making Test—part A; *TMT-B*, Trail Making Test—part B; *VFT-sum*, Verbal (Phenomic) Fluency Test—total cumulative number for three mora; *VFT-error*, Verbal (Phenomic) Fluency Test—number of repeated errors for the three mora

^a^Two-sample *t*-test

^b^Wilcoxon rank sum test

^c^Chi-square test

^*^p < 0.05

**Table 2:** Clinical characteristics in the cancer chemotherapy group

		T0	T1
Tumor stage	I	2	
	II	3	
	III	7	
	IV	7	
Cancer sites	Colorectum	5	
	Stomach	5	
	Esophagus	9	
Line of therapy	Adjuvant chemotherapy	8	
	Neoadjuvant chemotherapy	5	
	Chemotherapy	5	
	Chemoradiation	1	
Chemotherapy regimen	DCF	5	
	CAPOX	5	
	SOX + HER	2	
	SOX + NIVO	2	
	NIVO + IPI	2	
	S-1	1	
	CF	1	
	CF + NIVO	1	
Hemoglobin level	g/dL (SD) [95% CI]	12.31 (1.80)	11.74 (1.53)
		[11.44–13.18]	[10.97–12.50]
Co-occurring symptoms	Fatigue (%)	1 (5.2)	7 (36.8)
	Sleep disturbance (%)	0 (0)	4 (21.1)
	Pain (%)	0 (0)	1 (5.2)

*CAPOX*, capecitabine + oxaliplatin; *CF*, cisplatin + 5-FU; *CI*, confidence interval; *DCF*, docetaxel + cisplatin + 5-FU; *HER*, trastuzumab; *IPI*, ipilimumab; *NIVO*, nivolumab; *SD*, standard deviation; *SOX*, S-1 + oxaliplatin

**Fig. 1:**
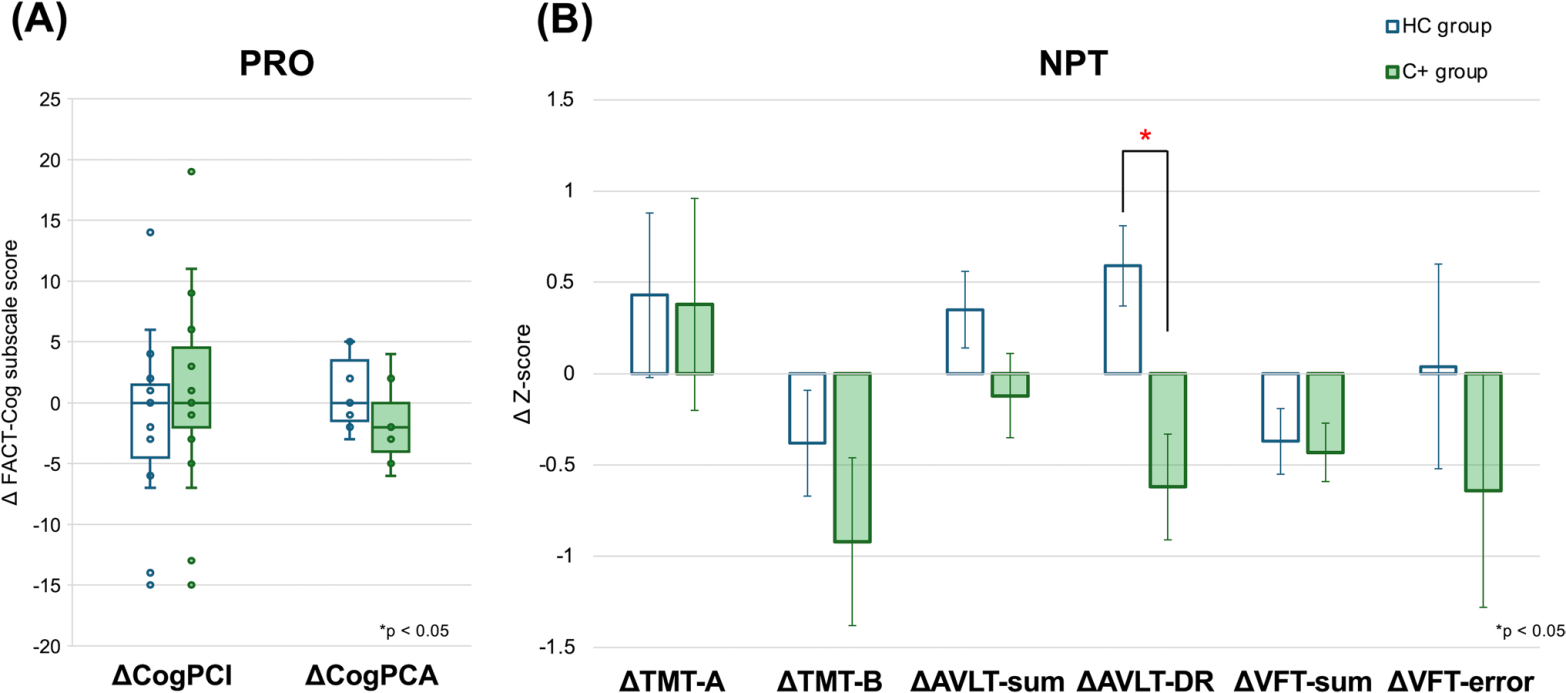
Between-group comparison of changes in cognitive assessment scores from baseline (T0) to 6 months follow-up (T1). **A** Patient-reported outcomes (PROs) using FACT-Cog. **B** Neuropsychological tests (NPTs). Error bars indicate standard error. The scores for all the assessment tools were positive for higher cognitive performance

Comparisons between the C + and HC groups at the baseline cognitive assessment showed no statistically significant difference in PRO and NPT scores (Table 1; Supplementary Table 1). The change in PRO and NPT between groups was not significantly different in the ΔCogPCI or ΔCogPCA scores on the FACT-Cog (Fig. 1A). A comparison of the NPT between the groups showed a statistically significant difference (*t* = 3.365, *p* = 0.002, *d* = 1.092) in the ΔAVLT-DR but no statistically significant change in the other NPT scores (Fig. 1B; Supplementary Table 2). As shown in Supplementary Fig. 1, the NPT scores showed an overall trend toward lower cognitive performance in the C + group than in the HC group. Within-group comparisons showed that the C + group had a significantly worse ΔAVLT-DR score (*t* = − 2.167, *p* = 0.044) compared to the baseline assessment. Conversely, the HC group showed a statistically significant improvement in the ΔAVLT-DR score (*t* = 2.710, *p* = 0.014) compared to the baseline assessment (Supplementary Table 3).

A comparison of the proportion of CRCI incidence by the difference in changes in PRO showed that the C + group was comparable (42.1%, *n* = 8) to the HC group (42.1%, *n* = 8) (Supplementary Fig. 2). Comparing the proportion of individuals with CRCIs using NPTs, the HC group showed a lower proportion of positive rates, 31.6% (*n* = 6), whereas the C + group showed a higher rate of 68.4% (*n* = 13) after chemotherapy (Supplementary Fig. 3). Comparing the proportions of subscales in between groups, the positive rates of AVLT-DR (31.6%, *n* = 6) and VFT errors (36.8%, *n* = 7) were higher in the C + group than in the HC group (Supplementary Fig. 3).

**Fig. 2:**
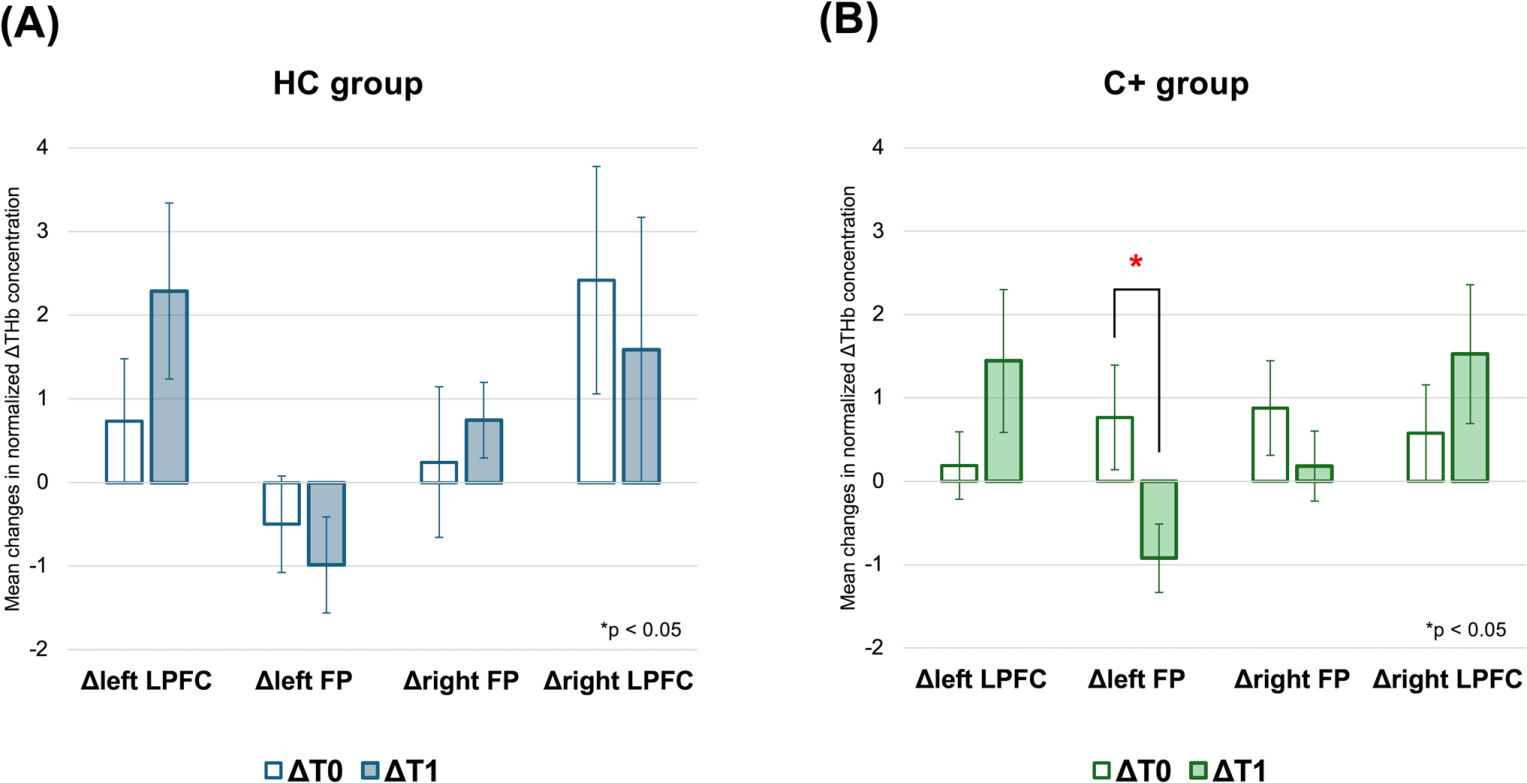
Within-group comparison of changes in total hemoglobin (THb) concentration in the prefrontal cortex. **A** Healthy control (HC) group. **B** Cancer chemotherapy (C+) group. Error bars indicate standard error. T0, baseline; T1, 6 months follow-up; LPFC, lateral prefrontal cortex; FP, frontal pole

As shown by the THb concentration change in Supplementary Fig. 4, a trend of increased task-dependent hemodynamic changes in the LPFC compared to the FP was observed in both groups according to the region of measurement. In terms of time-series changes, a trend toward a decrease in task-dependent hemodynamic changes at 6 months compared to baseline was observed in both groups. Task-dependent changes in THb concentrations were compared between and within groups, and no statistically significant differences were found in each channel in the between-group comparison (Supplementary Table 4). Within-group comparisons showed a main effect in the left FP region of the C + group (difference in change = − 1.6892; CI, − 3.0813 to − 0.2791; *p* = 0.018), with a significant reduction in THb concentration change at the 6-month follow-up assessment compared to pre-chemotherapy (Fig. 2; Supplementary Table 5).

## Discussion

This study investigated changes in subjective and objective cognitive function before and after chemotherapy, utilizing PROs, NPTs, and multidomain assessments that incorporated functional neuroimaging with a P-NIRS device. There were no significant differences in subjective cognitive complaints between the C + and HC groups. However, objective cognitive performance showed a reduction in delayed recall of verbal memory in the C + group. There was no difference between the groups in prefrontal cortical hemodynamic changes during the VFT task. The comparison within the C + group showed a decrease in the left FP cortical hemodynamics during chemotherapy compared to baseline.

The results of PRO using FACT-Cog showed no statistical difference between the two groups in the CogPCI and CogPCA scores, with minimal change within the groups. The proportion of CRCI cases in the C + group was 68.9% with the NPT assessment and 42.1% with the PRO assessment. Discrepancies between the subjective and objective assessments of the CRCI have been reported [37, 38], and the present study’s findings agree with those of previous studies. However, previous research on breast cancer survivors reported that cognitive complaints can be more sensitively assessed for CRCI [39]. The different results in the present study may be related to differences in sex and ethnicity, as patients with gastrointestinal cancer are more likely to be Japanese males. Many older Japanese men consider it undesirable to express their cognitive impairment to others. These sex differences and cultural contexts may suggest that PRO-dependent assessment is not always useful in CRCI studies of all cancers and objective assessment in combination with PRO.

Regarding the NPT results, the C + group showed statistically significant reductions in AVLT-DR scores compared to the HC group only in the verbal memory domain, with a significant worsening of AVLT-DR scores in the pre- and post-intervention comparisons within the C + group. There were no statistically significant differences in attentional function, processing speed, or executive function compared with the HC group, whereas some VFT performances were worse in the pre- and post-comparisons within the C + group. A meta-analysis of breast cancer survivors reported significant declines in delayed recall memory and executive function, even after accounting for baseline cognitive differences between the C + and HC groups [40]. On the other hand, a recent systematic review of colorectal cancer survivors showed that colorectal cancer treatment causes negligible changes in NPT performance, and delayed recall of verbal memory functions has a smaller effect size compared to other domains [41]. The present study showed different results, which may have been influenced by other gastrointestinal cancer diseases and differences in chemotherapy regimens. In addition, a previous study of Asian gastrointestinal cancer survivors showed that decreased attention, processing speed, memory, and executive function during chemotherapy were closely associated with fatigue [42]. The C + group had a higher rate of co-occurring symptoms, such as fatigue, than the pre-treatment group. However, considering that the cognitive domains impaired in this study were limited to delayed regeneration, with less reduction in attentional function and processing speed, which are closely associated with fatigue, it is suggested that the proportion of fatigue directly affecting cognitive function was small. Additional assessments using objective physiological markers, such as inflammatory markers, rather than subjective fatigue alone, could be a better way to clarify the association between CRCI and fatigue, and this is a limitation of this study.

Concerning the measurement of cortical hemodynamic changes using fNIRS, there were no notable differences between the C + and HC groups. A previous study using fNIRS assessment to capture CRCI in breast cancer survivors reported that LPFC hemodynamics and VFT performance were reduced in the chemotherapy treatment group compared to controls [19]. The difference with the present study is that Duran-Gomez et al. conducted a cross-sectional study that measured only the LPFC. Interestingly, the chemotherapy group in the present study showed decreased activity of the left anterior FP in the within-group comparisons. Generally, neuroimaging studies of VFT tasks have noted increased HbO_2_ and THb concentration changes in the LPFC over the FP during the VFT in healthy participants [43], and the left inferior frontal gyrus is most associated with word production during the VFT [44]. The FP is a cortical region involved in monitoring internally generated information during multitasking and episodic memory retrieval [45, 46]. In the present study, the C + group tended to have a higher left FP activity at baseline. These findings may indicate functional compensation for the temporarily necessary left FPs. Nevertheless, the small sample size requires careful interpretation of the results, and further studies are needed to show the relationship between VFT performance and cerebral hemodynamics in CRCI. Although this was a secondary finding, portable fNIRS has the potential to distinguish confounding factors due to motivational and depressive symptoms in patients with CRCI. In general, neurovascular coupling responses in the prefrontal cortex are significantly reduced in patients with depression [47]. The study’s findings indicate that the C + group showed at least similar or greater hemodynamic changes in the PFC than the HC group, and physiological parameters indicated that the cognitive decline was not due to depressive symptoms. Although depressive symptoms were not the primary focus of this study, continuous monitoring of physiological parameters throughout the study indicated that the cognitive decline may not be due to depressive symptoms. Future research that integrates cerebral hemodynamic monitoring during cognitive tasks with depression rating scale results, such as those derived from PRO, could further clarify the role of motivation and depressive symptoms in cognitive function assessments and improve their reliability.

This study had some limitations. First, these findings have low generalizability owing to selection biasassociated with recruiting the C + group from a single institution and the HC group from patients’ families and staff. In particular, family members or hospital staff may exhibit health behaviors or stress levels that differ from the general population, potentially confounding control group data. A community-based random sampling approach would have provided a more representative HC group. Second, the study’s fNIRS measurements focused solely on the prefrontal cortex, despite CRCI symptoms being linked to fronto-parietal networks and hippocampal-cortical connections [48, 49]. Measuring additional regions, such as the parietal or temporal lobes, might have yielded different results based on cognitive task conditions. The difficulty of capturing hippocampal activity with fNIRS also suggests that deeper brain measurements require alternative neuroimaging technologies. Finally, limitations in study design, including a small sample size and lack of follow-up data post-chemotherapy, restrict the ability to assess the effects of individual chemotherapy regimens. Additionally, this study lacks a comparison with a cancer survivor group who did not undergo chemotherapy (C − group). A meta-analysis of breast cancer survivors has shown differences in cognitive performance scores between the C + and HC groups, with a smaller decline in cognitive function when comparing C + to C − groups [39]. Future research should address this by employing a multidisciplinary assessment protocol in a multicenter, three-arm study (C + , C − , and HC groups) focusing on gastrointestinal cancer survivors to better understand the nuances of CRCI.

## Conclusion

In this prospective cohort study of cognitive changes in gastrointestinal cancer survivors, our findings showed that cancer survivors had lower delayed recall of memory function than healthy controls over 6 months. Furthermore, analysis of cognitive tasks using P-NIRS showed that cortical hemoglobin concentration changes differed before and after cancer chemotherapy. We believe that the evaluation of cognitive function combined with longitudinal portable NIRS is novel and sufficiently feasible for clinical application in the detection of CRCI.

## Supplementary Information

Below is the link to the electronic supplementary material.

Supplementary file1 (PDF 2737 KB)

## Data Availability

No datasets were generated or analysed during the current study.

## Abbreviations

CRCI: Cancer-related cognitive impairment
PRO: Patient-reported outcome
NVC: Neurovascular coupling
C +: Cancer chemotherapy
HC: Healthy control
VFT: Verbal fluency test
LPFC: Lateral prefrontal cortex
FACT-Cog: Functional Assessment of Cancer Therapy-Cognitive
ICCTF: International Cognition and Cancer Task Force
NPT: Neuropsychological tests
TMT: Trail making test
COWA: Controlled oral word association test
fMRI: Functional magnetic resonance imaging
fNIRS: Functional near-infrared spectroscopy
P-NIRS: Portable fNIRS
ECOG-PS: Eastern Cooperative Oncology Group-performance status
CogPCI: Perceived cognitive impairments
CogPCA: Perceived cognitive abilities
AVLT: Auditory verbal learning test
AVLT-DR: AVLT-delayed recall
THb: Total hemoglobin
FP: Frontal pole
