# Supplementary material for: Changes in chemotherapy-induced cognitive impairment in gastrointestinal cancer survivors using multidomain assessments: a prospective cohort study
Source: J Cancer Surviv. 2025 Feb 15;20(4):1601–11. doi: 10.1007/s11764-025-01759-8 (PMC13375846; doi:10.1007/s11764-025-01759-8)
Supplement: Supplementary file 1 — Supplementary file1 (PDF 2737 KB) [file 11764_2025_1759_MOESM1_ESM.pdf]

Supplementary Table 1. Between-group comparison of cognitive assessment at baseline and 6-month follow-up.

	Baseline			6-month follow-up		
	**C+ group (n=19)**	**HC group (n=19)**	**p-value**	**C+ group (n=19)**	**HC group (n=19)**	**p-value**
**Neuropsychological tests**	median (IQR) or mean [95%CI]					
TMT-A , sec	43 (33-74)	51 (34-59)	0.759[b]	42 (38-63)	42 (29-56)	0.381[b]
**TMT-A, z-score†**	**-1.26 [-2.49, 0.03]**	**-0.61 [-1.43, 0.20]**	**0.363[a]**	**-0.88 [-1.80, 0.04]**	**-0.18 [-0.97, 0.61]**	**0.232[a]**
TMT part B, sec	78 (59-119)	75 (67-98)	0.781[b]	93 (62-135)	89 (59-117)	0.381[b]
**TMT-B, z-score†**	**-0.99 [-2.12, 0.14]**	**-0.18 [-0.80, 0.43]**	**0.197[a]**	**-1.91 [-3.39, -0.44]**	**-0.56 [-1.37, 0.24]**	**0.102[a]**
AVLT-sum, words	37.11 [32.07, 42.14]	40.74 [35.93, 45.55]	0.281[a]	36.58 [32.34, 40.82]	43.84 [38.68, 49.00]	0.029[a]*
**AVLT-sum, z-score**	**-1.00 [-1.42, -0.59]**	**-0.58 [-1.10, -0.07]**	**0.190[a]**	**-1.12 [-1.60, -0.65]**	**-0.24 [-0.77, 0.30]**	**0.014[a]***
AVLT-DR, words	7.32 [5.65, 8.99]	7.84 [6.36, 9.33]	0.624[a]	5.74 [4.19, 7.28]	9.53 [7.92, 11.14]	0.001[a]**
**AVLT-DR, z-score**	**-0.78 [-1.33, -0.23]**	**-0.54 (0.23) [-1.01, -0.06]**	**0.482[a]**	**-1.41 [-1.97, -0.84]**	**0.06 [-0.54, 0.65]**	**<.001[a]****
VFT-sum, words	32.89 [27.83, 37.96)	32.79 [26.74, 38.84]	0.978[a]	32.89 [26.63, 39.16]	32.42 [27.10, 37.74]	0.904[a]
**VFT-sum, z-score**	**1.62 [0.97, 2.26]**	**1.26 [0.68, 1.85]**	**0.389[a]**	**1.62 [0.81, 2.43]**	**1.24 [0.63, 1.85]**	**0.427[a]**
VFT-error, words	2.42 [1.59, 2.42]	1.52 [0.34, 2.71]	0.202[a]	3.16 [1.87, 4.45]	1.47 [0.75, 2.20]	0.024[a]*
**VFT-error, z-score†**	**-1.32 [-1.98, -0.65]**	**-0.53 [-1.59, 0.53]**	**0.198[a]**	**-1.95 [-3.04, -0.87]**	**-0.49 [-1.15, 0.17]**	**0.022[a]***
**Patient-reported outcomes**	median (IQR)					
**FACT-Cog**						
**Cog PCI, score**	66 (59, 70)	65 (61, 68)	0.568[b]	68 (63-70)	65 (59-70)	0.223[b]
**Cog PCA, score**	24 (22, 26)	23 (22, 25]	0.385[b]	24 (16-27)	23 (21-23)	0.411[b]

AVLT-DR, (Rey) Auditory Verbal Learning Test - delayed recall (Trial 7); **AVLT-sum**, (Rey) Auditory Verbal Learning Test - sum (Trial 1-5); **C+ group**, Cancer survivor group with chemotherapy; **CI,** confidence interval; **Cog-PCA**, cognitive function – perceived cognitive ability; **Cog-PCI**, cognitive function – perceived cognitive impairment; **FACT-Cog**, Functional Assessment of Cancer Therapy - Cognitive Function; **HC group**, healthy control group; **IQR**, Interquartile range; **TMT-A**, Trail Making Test- part A; **TMT-B**, Trail Making Test - part B; **VFT-sum**, Verbal (Phenomic) Fluency Test - total cumulative number for three mora, **VFT-error**, Verbal (Phenomic) Fluency Test - number of repeated errors for the three mora

† The reversed values of the normalized score (Z-score) was used so that the positive direction of cognitive performance is better. a, two sample t-test; b, Wilcoxon rank sum test , *p<0.05 **p<0.01

Supplementary Table 2. Comparison of change in difference (Δ) for cognitive functional assessment between group

	C+ group (n=19)	HC group (n=19)	p-value
**Neuropsychological tests**	median (IQR) or mean [95%CI]		
Δ TMT-A , sec	-3 [-22, 15]	0 [-16, 5]	0.849[b]
**Δ TMT-A, score[†]**	0.38 [-0.84, 1.61]	0.43 [-0.52, 1.38]	**0.944[a]**
Δ TMT part B, sec	12 [-12, 30]	4 [-6, 10]	0.448[b]
**Δ TMT-B, score[†]**	-0.92 [-1.88, 0.04]	-0.38 [-0.99, 0.23]	**0.326[a]**
Δ AVLT-sum, words	-0.53 [-4.89, 3.83]	3.11 [-0.92, 7.13]	0.207[a]
**Δ AVLT-sum, score**	-0.12 [-0.61, 0.37]	0.35 [-0.10, 0.79]	**0.149[a]**
Δ AVLT-DR (Trial 7), words	-1.58 [-3.15, -0.01]	1.68 [0.44, 2.93]	0.002[a*]
**Δ AVLT-DR (Trial 7), score**	-0.62 [-1.23, -0.02]	0.59 [0.13, 1.05]	**0.002[a*]**
Δ VFT-sum, words	0 [-2.83, 2.83]	-0.37 [-3.30, 2.56]	0.850[a]
**Δ VFT-sum, score**	0.01 [-0.35, 0.37]	-0.01 [-0.36, 0.33]	**0.909[a]**
Δ VFT-error, words	0.74 [-0.92, 2.39]	-0.05 [-1.35, 1.25]	0.436[a]
**Δ VFT-error, score[†]**	-0.64 [-1.98, 0.70]	0.04 (0.56) [-1.14, 1.22]	**0.431[a]**
**Patient-reported outcomes**	median [IQR]		
**Δ FACT-Cog**			
**Δ Cog PCI, score**	0 [-3, 6]	0 [-6, 2]	0.356[b]
**Δ Cog PCA, score**	-1 [-3, 3]	0 [-2, 2]	0.770[b]

AVLT-DR, (Rey) Auditory Verbal Learning Test - delayed recall (Trial 7); **AVLT-sum**, (Rey) Auditory Verbal Learning Test - sum (Trial 1-5); **C+ group**, Cancer survivor group with chemotherapy; **CI,** confidence interval; **Cog-PCA**, cognitive function – perceived cognitive ability; **Cog-PCI**, cognitive function – perceived cognitive impairment; **FACT-Cog**, Functional Assessment of Cancer Therapy - Cognitive Function; **HC group**, healthy control group; **IQR**, Interquartile range; **SE**, standard error; **TMT-A**, Trail Making Test- part A; **TMT-B**, Trail Making Test - part B; **VFT-sum**, Verbal (Phenomic) Fluency Test - total cumulative number for three mora, **VFT-error**, Verbal (Phenomic) Fluency Test - number of repeated errors for the three mora

[†] The reversed values of the normalized score (Z-score) was used so that the positive direction of cognitive performance is better. a, two sample t-test; b, Wilcoxon rank sum test , *p<0.05

Supplementary Table 3. Within-group comparison of cognitive assessment in the C+ group and HC group

	**C+ group**			**HC group**		
	**Baseline**	**6-month follow-up**	**p-value**	**Baseline**	**6-month follow-up**	**p-value**
**Neuropsychological tests**	median (IQR) or mean [95%CI]					
TMT-A , sec	43 (33-74)	42 (38-63)	0.459[e]	51 (34-59)	42 (29-56)	0.487[e]
**TMT-A, z-score†**	**-1.26 [-2.49, 0.03]**	**-0.88 [-1.80, 0.04]**	**0.523[d]**	**-0.61 [-1.43, 0.20]**	**-0.18 [-0.97, 0.61]**	**0.353[d]**
TMT part B, sec	78 (59-119)	93 (62-135)	0.085[e]	75 (67-98)	89 (59-117)	0.378[e]
**TMT-B, z-score†**	**-0.99 [-2.12, 0.14]**	**-1.91 [-3.39, -0.44]**	**0.058[d]**	**-0.18 [-0.80, 0.43]**	**-0.56 [-1.37, 0.24]**	**0.204[d]**
AVLT-sum, words	37.11 [32.07, 42.14]	36.58 [32.34, 40.82]	0.803[d]	40.74 [35.93, 45.55]	43.84 [38.68, 49.00]	0.122[d]
**AVLT-sum, z-score**	**-1.00 [-1.42, -0.59]**	**-1.12 [-1.60, -0.65]**	**0.611[d]**	**-0.58 [-1.10, -0.07]**	**-0.24 [-0.77, 0.30]**	**0.123[d*]**
AVLT-DR, words	7.32 [5.65, 8.99]	5.74 [4.19, 7.28]	0.049[d*]	7.84 [6.36, 9.33]	9.53 [7.92, 11.14]	0.011[d*]
**AVLT-DR, z-score**	**-0.78 [-1.33, -0.23]**	**-1.41 [-1.97, -0.84]**	**0.044[d*]**	**-0.54 (0.23) [-1.01, -0.06]**	**0.06 [-0.54, 0.65]**	**0.014[d*]**
VFT-sum, words	32.89 [27.83, 37.96)	32.89 [26.63, 39.16]	1.000[d]	32.79 [26.74, 38.84]	32.42 [27.10, 37.74]	0.794[d]
**VFT-sum, z-score**	**1.62 [0.97, 2.26]**	**1.62 [0.81, 2.43]**	**0.981[d]**	**1.26 [0.68, 1.85]**	**0.90 [0.31, 1.49]**	**0.933[d]**
VFT-error, words	2.42 [1.59, 2.42]	3.16 [1.87, 4.45]	0.361[d]	1.52 [0.34, 2.71]	1.47 [0.75, 2.20]	0.933[d*]
**VFT-error, z-score†**	**-1.32 [-1.98, -0.65]**	**-1.95 [-3.04, -0.87]**	**0.331[d]**	**-0.53 [-1.59, 0.53]**	**-0.49 [-1.15, 0.17]**	**0.942[d*]**
**Patient-reported outcomes**	median (IQR)					
**FACT-Cog**						
**Cog PCI, score**	66 (59, 70)	68 (63-70)	0.605[e]	65 (61, 68)	65 (59-70)	0.364[e]
**Cog PCA, score**	24 (22, 26)	24 (16-27)	0.641[e]	23 (22, 25]	23 (21-23)	0.921[e]

AVLT-DR, (Rey) Auditory Verbal Learning Test - delayed recall (Trial 7); **AVLT-sum**, (Rey) Auditory Verbal Learning Test - sum (Trial 1-5); **C+ group**, Cancer survivor group with chemotherapy; **CI,** confidence interval; **Cog-PCA**, cognitive function – perceived cognitive ability; **Cog-PCI**, cognitive function – perceived cognitive impairment; **FACT-Cog**, Functional Assessment of Cancer Therapy - Cognitive Function; **HC group**, healthy control group; **IQR**, Interquartile range; **TMT-A**, Trail Making Test- part A; **TMT-B**, Trail Making Test - part B; **VFT-sum**, Verbal (Phenomic) Fluency Test - total cumulative number for three mora, **VFT-error**, Verbal (Phenomic) Fluency Test - number of repeated errors for the three mora

† The reversed values of the normalized score (Z-score) was used so that the positive direction of cognitive performance is better. d, paired t-test; e, Wilcoxon sign rank test , *p<0.05 **p<0.01

Supplementally Table 4 : Comparison of mean changes in THb concentration between group

	Channel	Cancer with chemotherapy group			Healthy control group			t-value	p-value
		On control task (95% CI)	On VFT task (95% CI)	Change (95% CI)	On control task (95% CI)	On VFT task (95% CI)	Change (95% CI)		
T0	Left LPFC	1.009 (0.1839 to 1.8336)	1.1987 (0.5956 to 1.8017)	0.1899 (0.3782 to 0.9844)	0.8167 (-0.0947 to 1.7280)	1.5513 (0.3616 to 2.7410)	0.7347 (-0.7215 to 2.1908)	-0.69	0.4947
	Left FP	-0.0561 (-1.2689 to 1.1567)	0.7095 (-0.1607 to 1.5797)	0.7657 (-0.4619 to 1.9932)	0.6791 (-0.4433 to 1.8016)	0.1812 (-0.6424 to 1.0048)	-0.4979 (-1.6291 to 0.6333)	1.59	0.1205
	Right FP	-0.1777 (-0.8198 to 0.4644)	0.70055 (-0.6084 to 2.0095)	0.8783 (-0.2300 to 1.9865)	0.3667 (-0.3202 to 1.0536)	0.6103 (-1.1078 to 2.3283)	0.2435 (-1.5136 to 2.0059)	0.64	0.5250
	Right LPFC	0.7326 (0.2098 to 1.2553)	1.3130 (0.1432 to 2.4827)	0.5804 (-0.5451 to 1.7059)	0.4647 (-0.1234 to 1.0528)	2.8827 (0.3669 to 5.3985)	2.4180* (-0.2497 to 5.0857)	-1.33	0.1908
T1	Left LPFC	-0.4252 (-1.2596 to 0.4091)	1.0187 (-0.7817 to 2.8190)	1.4439 (-0.2342 to 3.1220)	0.1734 (-0.6237 to 0.9705)	2.4616 (0.6962 to 4.2271)	2.2882* (0.2235 to 4.3529)	-0.67	0.5092
	Left FP	0.8049 (-0.2774 to 1.8873)	-0.1186 (-0.7883 to 0.5511)	-0.9235* (-1.7287 to -0.1184)	1.1348 (-0.0165 to 2.2862)	0.1492 (-0.4381 to 0.7364)	-0.9857* (-0.1831 to -0.1404)	0.11	0.9118
	Right FP	-0.207 (-0.9780 to 0.5641)	-0.0235 (-1.2121 to 1.1651)	0.1835 (-0.6412 to 1.0082)	0.0984 (-0.4957 to 0.6924)	0.8431 (-0.2202 to 1.9065)	0.7448 (-0.1366 to 1.6262)	-0.97	0.3371
	Right LPFC	-0.4671 (-1.3109 to 0.3767)	1.0586 (-0.6102 to 2.7274)	1.5257 (-0.1052 to 3.1566)	0.2076 (-0.5011 to 0.9163)	1.7924 (-1.2426 to 4.8274)	1.5848 (-1.52325 to 4.6928)	-0.04	0.9720

Values are mean (95% confidence interval), T0: pre-chemotherapy or baseline assessments, T1: six-months follow-up assessments, FP: Frontal pole, LPFC: Lateral prefrontal cortex, VFT: verbal fluency test

Supplemental Table 5: Comparison of mean changes in THb concentration within groups

	Ch	T0: pre-chemotherapy			T1: 6-month follow-up			Difference in change (95% CI)	P-value
		Baseline (control task) (95% CI)	On task (95% CI)	Change (95% CI)	Baseline (95% CI)	On task (95% CI)	Change (95% CI)		
C+	Left LPFC	1.009<br>(0.1839 to 1.8336)	1.1987<br>(0.5956 to 1.8017)	0.1899<br>(0.3782 to 0.9844)	-0.4252<br>(-1.2596 to 0.4091)	1.0187<br>(-0.7817 to 2.8190)	1.4439<br>(-0.2342 to 3.1220)	1.2540<br>(-0.7115 to 3.2196)	0.2063
	Left FP	-0.0561<br>(-1.2689 to 1.1567)	0.7095<br>(-0.1607 to 1.5797)	0.7657<br>(-0.4619 to 1.9932)	0.8049<br>(-0.2774 to 1.8873)	-0.1186<br>(-0.7883 to 0.5511)	-0.9235*<br>(-1.7287 to -0.1184)	-1.6892<br>(-3.0813 to -0.2971)	0.0184*
	Right FP	-0.1777<br>(-0.8198 to 0.4644)	0.70055<br>(-0.6084 to 2.0095)	0.8783<br>(-0.2300 to 1.9865)	-0.207<br>(-0.9780 to 0.5641)	-0.0235<br>(-1.2121 to 1.1651)	0.1835<br>(-0.6412 to 1.0082)	-0.6948<br>(-2.2113 to -0.8217)	0.3619
	Right LPFC	0.7326<br>(0.2098 to 1.2553)	1.3130<br>(0.1432 to 2.4827)	0.5804<br>(-0.5451 to 1.7059)	-0.4671<br>(-1.3109 to 0.3767)	1.0586<br>(-0.6102 to 2.7274)	1.5257<br>(-0.1052 to 3.1566)	0.9453<br>(-1.0643 to 2.9549)	0.3498
		T0: baseline			T0: 6-month follow-up				
HC	Left LPFC	0.8167<br>(-0.0947 to 1.7280)	1.5513<br>(0.3616 to 2.7410)	0.7347<br>(-0.7215 to 2.1908)	0.1734<br>(-0.6237 to 0.9705)	2.4616<br>(0.6962 to 4.2271)	2.2882*<br>(0.2235 to 4.3529)	1.5535<br>(-0.5674 to 3.6745)	0.1478
	Left FP	0.6791<br>(-0.4433 to 1.8016)	0.1812<br>(-0.6424 to 1.0048)	-0.4979<br>(-1.6291 to 0.6333)	1.1348<br>(-0.0165 to 2.2862)	0.1492<br>(-0.4381 to 0.7364)	-0.9857*<br>(-0.1831 to -0.1404)	-0.4878<br>(-1.8637 to 0.8882)	0.4803
	Right FP	0.3667<br>(-0.3202 to 1.0536)	0.6103<br>(-1.1078 to 2.3283)	0.2435<br>(-1.5136 to 2.0059)	0.0984<br>(-0.4957 to 0.6924)	0.8431<br>(-0.2202 to 1.9065)	0.7448<br>0.9844(-0.1366 to 1.6262)	0.5013<br>(-1.3138 to 2.3163)	0.5821
	Right LPFC	0.4647<br>(-0.1234 to 1.0528)	2.8827<br>(0.3669 to 5.3985)	2.4180*<br>(-0.2497 to 5.0857)	0.2076<br>(-0.5011 to 0.9163)	1.7924<br>(-1.2426 to 4.8274)	1.5848<br>(-1.52325 to 4.6928)	-0.8332<br>(-4.4224 to 2.7560)	0.6435

Values are mean (95% confidence interval), T0: pre-chemotherapy or baseline assessments, T1: six-months follow-up assessments, FP: Frontal pole, LPFC: Lateral prefrontal cortex, VFT: verbal fluency test

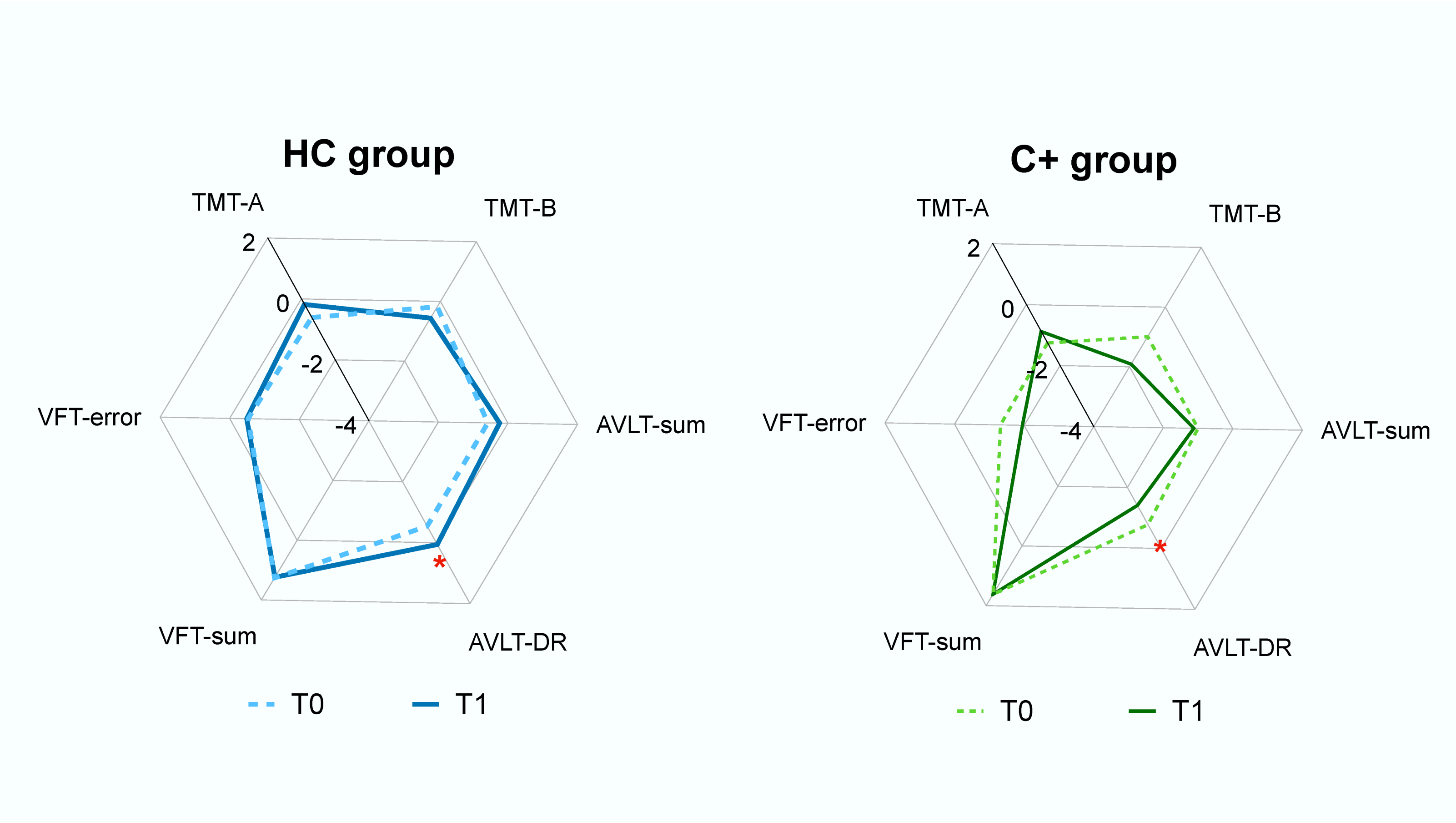

Supplementary Figure 1. Comparisons of neuropsychological tests in within-group

The normalized Z-scores for all assessment tools were positive for higher cognitive performance (*p<0.05).

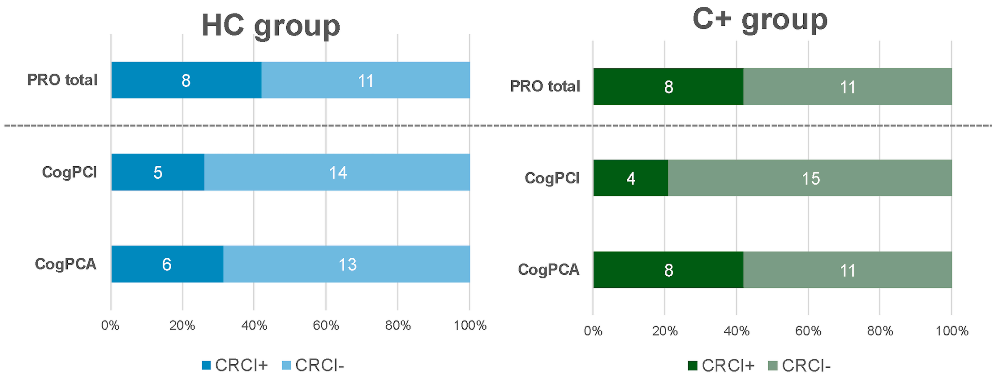

Supplementary Figure 2. CRCI incidence in C+ group and HC group using FACT-Cog subscales

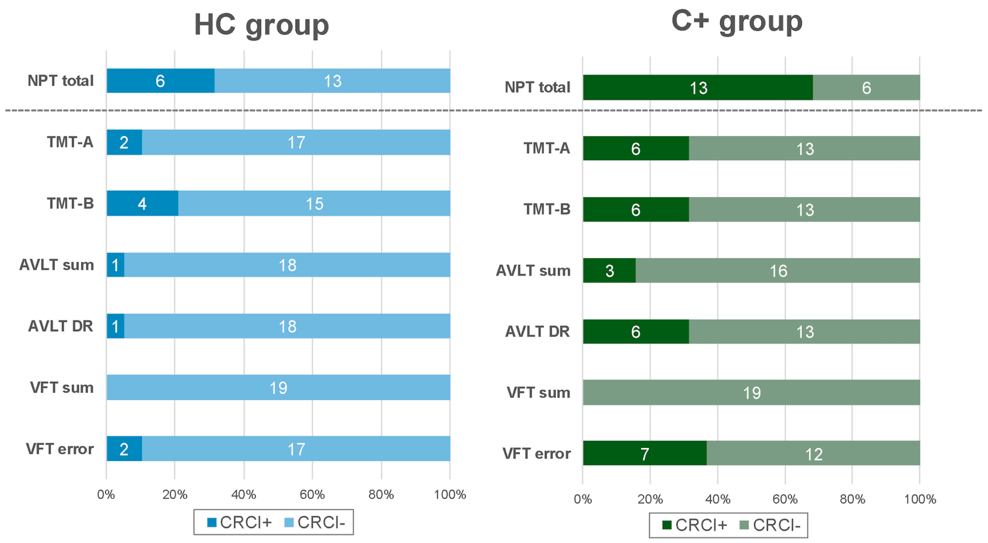

Supplementary Figure 3. CRCI incidence in C+ group and HC group using NPT subscales

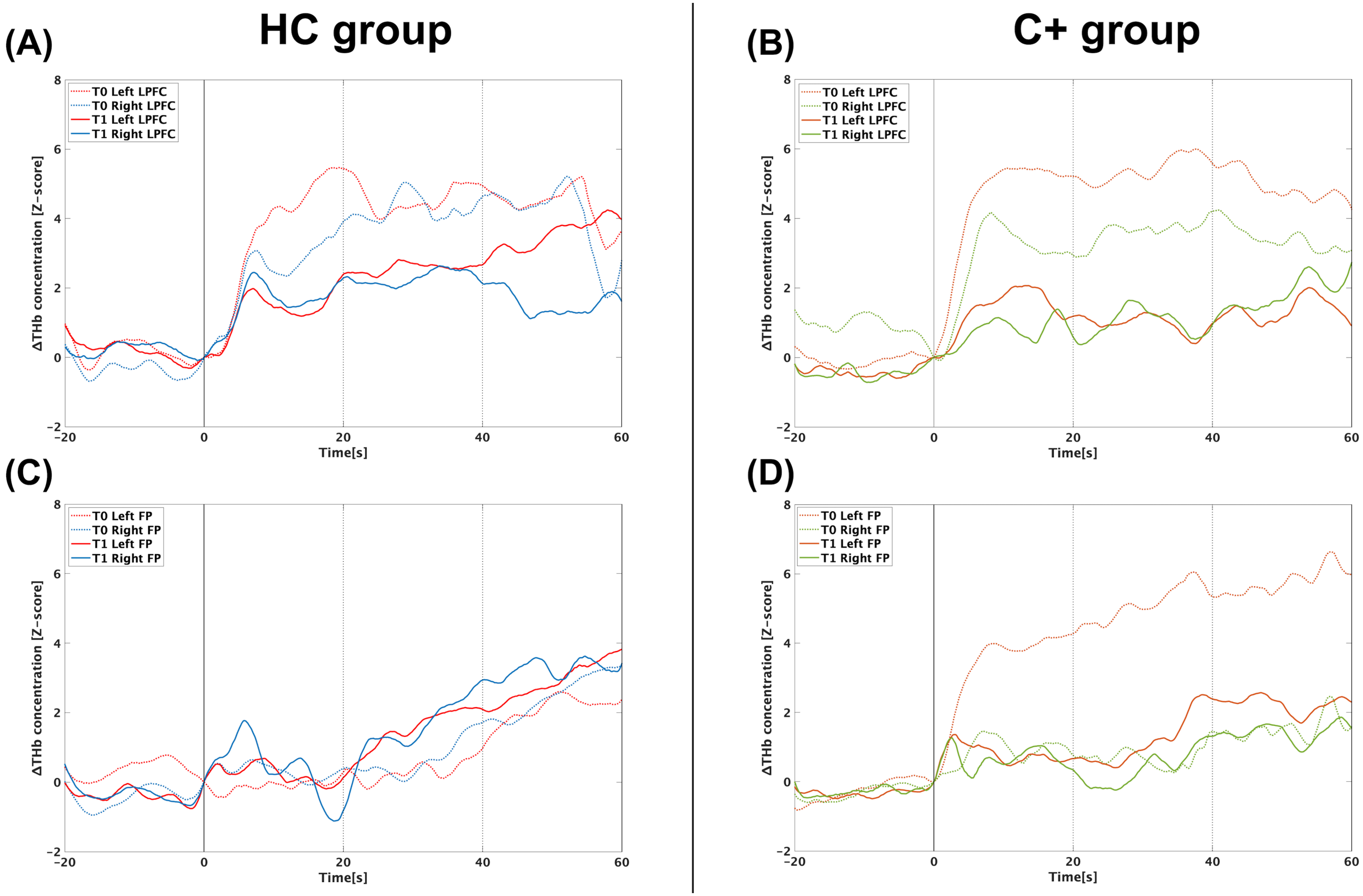

Supplementary Figure 4. The waveforms of total Hb concentration changes in both lateral prefrontal cortex (LPFC) and frontal pole (FP) regions during cognitive tasks in baseline (T0) and follow-up (T1) assessments. A) Right LPFC, B) Left LPFC, C) Right FP, D) Left FP.
